# Transposable elements in a clade of three tetraploids and a diploid relative, focusing on Gypsy amplification

**DOI:** 10.1186/s13100-015-0034-8

**Published:** 2015-03-25

**Authors:** Mathieu Piednoël, Aretuza Sousa, Susanne S Renner

**Affiliations:** Systematic Botany and Mycology, University of Munich (LMU), Botanisches Institut, Menzinger Str. 67, Munich, 80638 Germany; Department of Plant Developmental Biology, Max-Planck Institute for Plant Breeding Research, Carl-von-Linne-Weg 10, Cologne, 50829 Germany

**Keywords:** Transposable elements, Ploidy, Chromosomes, Gypsy, Copia, *Orobanche*

## Abstract

**Background:**

Polyploidization can activate specific transposable elements, leading to their accumulation. At the same time, the preferential loss of repetitive elements in polyploids may be central to diploidization. The paucity of studies of transposable element (TE) dynamics in closely related diploid and polyploid species, however, prevents generalizations about these patterns. Here, we use low-coverage Illumina sequencing data for a clade of three tetraploid *Orobanche* species and a diploid relative to quantify the abundance and relative frequencies of different types of TEs. We confirmed tetraploidy in the sequenced individuals using standard cytogenetic methods and inferred the time of origin of the tetraploid clade with a rate-calibrated molecular clock.

**Findings:**

The sequenced individuals of *Orobanche austrohispanica*, *Orobanche densiflora*, and *Orobanche gracilis* have 2*n* = 76 chromosomes, are tetraploid, and shared a most recent common ancestor some 6.7 Ma ago. Comparison of TE classifications from the Illumina data with classification from 454 data for one of the species revealed strong effects of sequencing technology on the detection of certain types of repetitive DNA. The three tetraploids show repeat enrichment especially of Gypsy TE families compared to eight previously analyzed Orobanchaceae. However, the diploid *Orobanche rapum-genistae* genome also has a very high proportion (30%) of Gypsy elements.

**Conclusions:**

We had earlier suggested that tetraploidization might have contributed to an amplification of Gypsy elements, particularly of the Tekay clade, and that *O. gracilis* underwent genome downsizing following polyploidization. The new data reveal that Gypsy amplification in Orobanchaceae does not consistently relate to tetraploidy and that more species sampling is required to generalize about Tekay accumulation patterns.

**Electronic supplementary material:**

The online version of this article (doi:10.1186/s13100-015-0034-8) contains supplementary material, which is available to authorized users.

## Findings

### Background

The effects of transposable elements (TEs) on genome size can be highly unpredictable [1]. While some plant lineages, even with ancient whole-genome duplication, have undergone rapid DNA removal and show no TE amplification ([2]: *Gossypium*; [3]: *Oryza brachyantha*), others have experienced dramatic genome growth because of high levels of TE amplification [4]. An open question is the cause of such different levels of genome stability or instability. To answer this question, in-depth analyses are required of closely related populations or species that differ only in factors suspected to affect TE accumulation. Among the factors known to activate transposable elements are hybridization and polyploidization, which in plants are often associated with each other. Several studies have demonstrated that polyploidy can activate specific transposable elements, translating into bursts of transposition [5,6]. The dynamics of TEs in allopolyploid species of *Nicotiana*, associated with genome turnover over the past 5 million years, at the same time suggests that repetitive elements are central to the diploidization process [6-9]. The paucity of studies of the TE dynamics in closely related diploid and polyploid species, however, prevents generalizations about possible patterns of bursts in TE activation and accumulation in polyploids [1].

In two earlier studies, we characterized the repetitive DNA content of a tetraploid species, *Orobanche gracilis*, in comparison to eight diploid Orobanchaceae from four genera, including *Orobanche* [10,11]. We suggested that tetraploidization might have contributed to a transposition burst of Gypsy TE families specific to *O. gracilis*, particularly of the Tekay clade. This clade contains some of the largest chromoviruses [12], and its elements are hypothesized to preferentially insert in heterochromatin [13]. We also found that *O. gracilis* lost several repeat families widely shared among diploid Orobanchaceae and that it has a relatively small genome. We therefore hypothesized that these families might have been lost during genome downsizing following polyploidization.

To test these hypotheses about the effects of polyploidization on TE dynamics in Orobanchaceae, we selected two other polyploids, *Orobanche austrohispanica* and *Orobanche densiflora*, whose geographic ranges partially overlap with that of *O. gracilis*, although the three species are not known to co-occur at the local scale or to hybridize. As outgroup, we included a representative of their diploid sister clade, *Orobanche rapum-genistae* [10,14]. We obtained paired-end Illumina sequence data for all four species and then studied their repeats using bioinformatics approaches. Our previous analysis of *O. gracilis* had relied on 454 pyrosequencing data rather than Illumina reads. This circumstance permitted us to test for artifacts of sequencing technology.

### Methods

#### Plant material

*Orobanche austrohispanica* M.J.Y. Foley is restricted to Southern Iberian Peninsula and to Morocco. It mostly parasitizes species of *Ulex* and other Fabaceae. The studied individual was collected at San Roque in Cadiz province, Andalucía, Spain, where it was parasitizing *Stauracanthus genistoides* (Brot.) Samp (voucher: M. V. Silber 13; Munich herbarium). *O. gracilis* Sm. is distributed in the Mediterranean northward to southern Central Europe. It parasitizes shrubby Fabaceae. The sequenced plant was collected at the Botanical Garden of Munich (Germany) where it was parasitizing *Genista tinctoria* L. (voucher: M. V. Silber 17; Munich herbarium). *O. densiflora* Salz. ex Reut. is distributed from the Iberian Peninsula to the Maghreb in western North Africa. It parasitizes Fabaceae and Asteraceae. The sequenced plant was collected at La Linea de la Concepción in Cadiz province, where it was parasitizing *Lotus creticus* L. (voucher: M. V. Silber 12; Munich herbarium). *O. rapum-genistae* Thuill. is widely distributed in Western Europe. It parasitizes *Ulex europaeus* L., *Cytisus scoparius* (L.) Link, and other woody Fabaceae. The sequenced plant was collected at Mook-Molenhoek, near the train track from Nijmegen to Venlo, where it was parasitizing *C. scoparius* (voucher: Bert Kapteyn s.n.; Munich herbarium).

#### DNA isolation and Illumina sequencing

**Figure 1:**
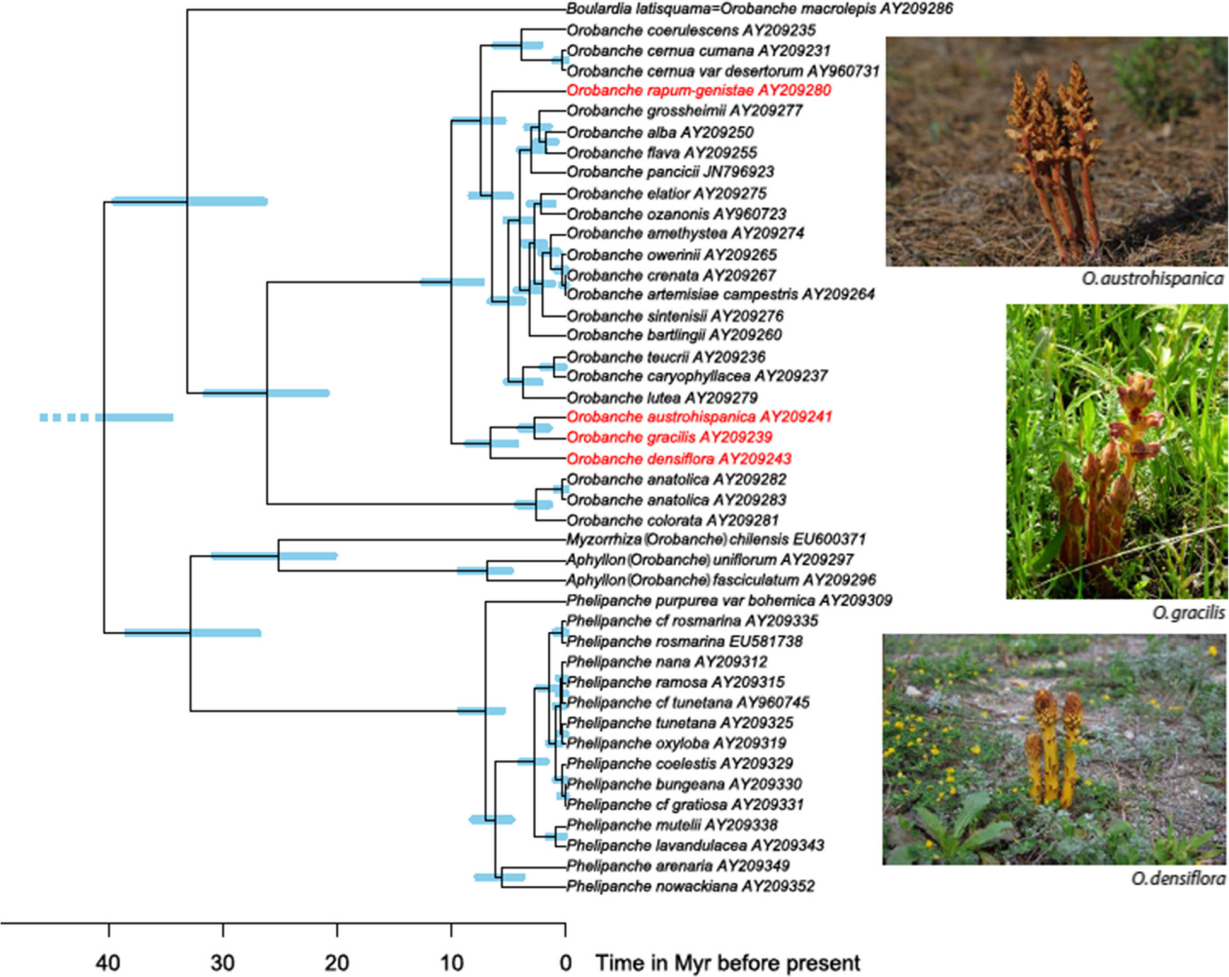
**Chronogram for the Orobanchaceae.** Based on nuclear rDNA internal transcribed spacer sequences, with the tetraploid focal clade and its diploid outgroup highlighted in red. Habit photos of *Orobanche austrohispanica* and *O. densiflora* by J. A. Garcia Rojas, *O. gracilis* by S. S. Renner.

DNA isolation relied on the Qiagen (Hilden, Germany) DNeasy Plant Maxi Kit, using about 5 g of fresh flower material (these parasitic plants have no green leaves; see photos in Figure 1). For each species, approximately 7 μg of genomic DNA were submitted for sequencing at GATC Biotech (Konstanz, Germany), with libraries prepared according to Illumina instructions and sequencing performed on the Genome Analyzer II platform (Illumina, San Diego CA, USA), for 101-base pair-long paired-end reads.

#### Genome size estimation and chromosome preparation

The *C* values of two individuals of *O. densiflora* and one individual of *O. austrohispanica* were measured using flow cytometry with propidium iodide (PI) as the DNA stain and *Solanum pseudocapsicum* as the standard, following the protocol of Temsch *et al.* [15]. Fresh material was co-chopped together with the standard plant in Otto’s buffer I. The resulting suspension was filtered (30-μm nylon mesh), RNase treated, and incubated in PI containing Otto’s buffer II. A CyFlow ML flow cytometer (Partec, Muenster, Germany) equipped with a green laser (100 mW, 532 nm, Cobolt Samba, Cobolt, Stockholm, Sweden) was used for the fluorescence measurements, with 5,000 particles measured per run and three runs performed per plant preparation. The *C* value was calculated according to the formula: 1*C* value_Object_ = (mean G1 nuclei fluorescence intensity_Object_/mean G1 nuclei fluorescence intensity_Standard_)*1*C* value_Standard_. The peak CV percentages usually were <5%.

Meiotic cell preparations were made from anthers of young buds. Anthers were fixed in 3:1 (*v*/*v*) ethanol/glacial acetic acid at room temperature overnight and stored at −20°C. Fixed anthers were quickly washed in distilled water and dissected in a drop of 45% acetic acid and squashed. Coverslips were removed after freezing, and slides were air-dried at room temperature before being stained with DAPI (2 μg/ml) mounted in Vectashield (Vector Laboratories, Peterborough, UK). As buds of *O. austrohispanica*, *O. densiflora*, and *O. gracilis* had been collected at different times, we used both meiotic and pro-meiotic cells to access their ploidy levels. Images were taken with a Leica DMR microscope (Leica, Solms, Germany) equipped with a KAPPA-CCD camera (Kappa, Gleichen, Germany) and the KAPPA software. They were optimized for best contrast and brightness using Adobe Photoshop CS3 version 10.0 (Adobe, San Jose, CA, USA).

#### Phylogenetic placement and molecular clock dating

To place the study species in a phylogenetic context, we obtained nuclear internal transcribed spacer sequences (including the 5.8S gene) from the Illumina data and added them to a large internal transcribed spacer (ITS) matrix built for previous studies [10,14,16]. Tree searching and bootstrapping (with 100 replicates) relied on maximum likelihood under the general time reversible (GTR) + G model of substitution, using RAxML version 7.2.8 [17]. Clock dating was done in BEAST v. 1.8 [18], using the GTR + Γ substitution model with six rate categories, a pure-birth (Yule) tree prior, a strict clock model, and an MCMC chain length between 10 and 20 million generations, sampling every 10,000th generation, with convergence determined by examining the log files in Tracer v. 1.5 (Rambaut and Drummond, [19]) after removal of a burn-in proportion of 10% of the trees. The genetic distances were calibrated with an ITS substitution rate of 4.52 substitutions/site/year × 10^−9^ from the herbaceous Gentianaceae *Gentianella* [20].

#### Repeat identification

Raw sequencing data were preprocessed as follows: (i) remaining adapters were removed using Cutadapt (https://github.com/marcelm/cutadapt), (ii) quality and length filtering were performed using Sickle (Joshi and Fass, [21]) with the -q 20 -l 100 -n parameters, and (iii) duplicates were removed using FastUniq [22]. A combined dataset comprising 300,000 read pairs randomly sampled from each species was used to characterize genomic repeats. In addition, an individual dataset for *O. gracilis* was designed by randomly sampling 900,000 of its Illumina sequencing read pairs. Highly and moderately repetitive sequences were identified using RepeatExplorer [23], a similarity-based clustering tool for next-generation sequencing reads. A similarity cut-off of 90% over at least 80% of the read length was used for the clustering, and the reads within individual clusters were assembled. Annotation of the repeats relied on RepeatMasker [24] and the MIPS Repeat Element Database (MIPS REdat; [25]) as well as similarity searches (BLASTn and BLASTx; *E* value threshold e-15) against GenBank [26]. Additional tBLASTx analyses were performed against MIPS REdat (*E* value threshold e-15), and conserved protein domains were detected using RPS-tBLASTn (*E* value threshold e-5). Satellites were found with Tandem Repeat Finder [27] and by their particular features in similarity-graph visualization [28]. All repeat families were manually annotated using the results of the different programs, with priority given to BLASTn and BLASTx results, except when one of the other programs yielded much lower *E* values. Connections between clusters due to split read pairs were used to confirm and/or infer annotations. Copia and Gypsy retrotransposons were further classified into previously described clades [29-31] using similarity searches against the GypsyDatabase [32]. Families were assigned to clades only if all their best hits exceeded a minimum *E* value of 10–5. The Copia clades Bianca and Tos17 are lacking in the GypsyDatabase and were therefore not detected here. Two other Copia clades, Angela and TONT1, not represented in the GypsyDatabase are closely related to the Tnt1 clade, and we consider all three clades as members of a class called ‘Tnt1-Angela-TONT1.’

### Results and discussion

#### Molecular clock dating, chromosome numbers, and genome sizes

Figure 1 shows a chronogram for the Orobanchaceae, with the focal species’ highlighted in red. The crown group age of the parasitic Orobanchaceae in our tree is 40 (35 to 46) Ma (Figure 1), which overlaps with the only other published estimate for this clade of 32 (13 to 52) Ma by Naumann *et al.* [33]. In the absence of a fossil record, it is difficult to judge these estimates. However, the inferred age of 6.67 (4.4 to 8.5) Ma for the most recent common ancestor of the three tetraploid species appears reasonable, given their small morphological differences (see photos in Figure 1).

**Figure 2:**
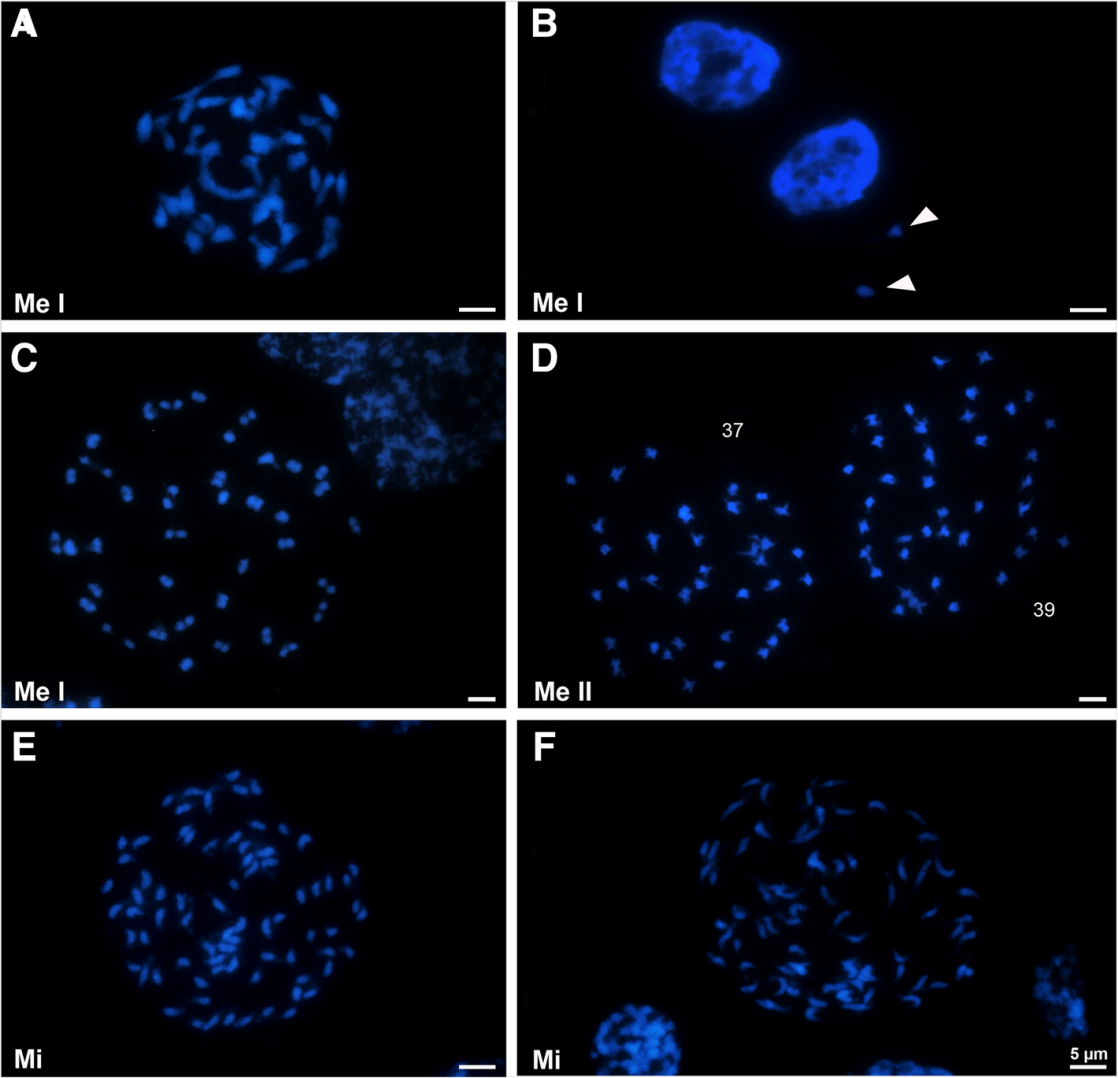
**Meiotic (Me I or II) and mitotic (Mi) cells stained with DAPI. (A)** Prophase I of *Orobanche austrohispanica* with ca. 38 bivalents (2*n* = 4*x* = 76). **(B)** Interphase nuclei of same species with two micronuclei (arrowheads). **(C)** Prophase I of *O. densiflora* with 38 bivalents (2*n* = 4*x* = 76). **(D)** Metaphase II of same species with unbalanced number of chromosomes in each nucleus (37 and 39). **(E)** and **(F)** metaphase plates with condensed and more distended chromosomes of *O. gracilis* with 2*n* = 4*x* = 76. Bars correspond to 5 μm.

Several cells from one individual of *O. austrohispanica* and *O. densiflora* were newly karyotyped (Figure 2); chromosome numbers for *O. rapum-genistae* and *O. gracilis* were available [34]. Metaphase profiles of the two newly karyotyped species are shown in Figure 2. While *O. rapum-genistae* has 2*n* = 38 chromosomes, the three other species have 2*n* = 76 chromosomes and are tetraploid. Based on the phylogeny (Figure 1), tetraploidy stems from a single polyploidization event. The genome size of *O. austrohispanica* is 1*C* = 2.35 pg (one individual measured), of *O. densiflora* 1*C* = 2.17 to 2.20 pg (two individuals), of *O. gracilis* 2.10 pg [10], and of *O. rapum-genistae* 2.57 pg [35]. In base pairs, these species thus have genomes ranging from 2.05 Gb for *O. gracilis* to 2.5 Gb for *O. rapum-genistae*, with the diploid *O. rapum-genistae* having a slightly larger genome than the three tetraploid species. All these genome sizes are similar to those of other *Orobanche* [35].

#### Comparison of Illumina paired-end sequencing and 454 single-end pyrosequencing data as regards repeat DNA analysis

Illumina sequencing for this study returned from 76,525,545 to 137,063,530 pairs of 101-bp reads per species, resulting in 85.9 Gb of sequencing data (15.5 to 27.7 Gb per species). Differences between 454 and Illumina sequencing methodologies (in sequencing accuracy, read length, single-end *vs.* paired-end) may affect repeat identification. To assess the extent of such effects, we classified the repeats of *O. gracilis* with the new Illumina data and compared the results with our earlier repeat classification from 454 data (based on 350,000 reads of 300 bp; [10]). To do this, we subjected 900,000 high-quality Illumina read pairs from *O. gracilis* to RepeatExplorer (Materials and Methods), which partitioned the data into groups of overlapping reads representing individual repeat families. The read dataset corresponds to 0.088X coverage of the *O. gracilis* genome, higher than in our previous study. The detailed results obtained for each repeat type, as well as the earlier 454-based results, are shown in Table 1. The total TE content is higher using the Illumina dataset (68.68%) compared to the 454 dataset (60.13%), mostly due to the identification of satellites. While satellites make up only 5.08% of the *O. gracilis* genome with the 454 data, they make up 14.41% with the Illumina data. The difference in sequencing error rates and the sensitivity of 454 pyrosequencing to homo-polymer sequence miscalls [36] probably explain this difference. While the estimated rRNA fraction is hardly affected by sequencing method, three TE superfamilies were greatly affected: Gypsy retrotransposons were better detected with the 454 data (28.34%) compared to the Illumina data (23.64%), while Copia elements and hAT transposons were better detected with the Illumina data (21.63% and 1.40%, respectively) compared to the 454 sampling (18.41% and 0.11%, respectively).

**Table 1:** **Repeat composition of** ***Orobanche*** **species**

	**454**				**Illumina**					
	**Ocum**	**Ocre**	**Opan**	**Ogra**	**Ogra (IND)**	**Ogra (CD)**	**Oden (CD)**	**Oaus (CD)**	**Orap (CD)**	**Orap (IND)**
Gypsy	*17.02*	*21.44*	*24.16*	*28.34*	23.64	27.62	36.25	39.64	30.47	27.62
Copia	*16.01*	*21.42*	*18.82*	*18.41*	21.63	20.52	22.00	20.15	16.06	13.89
Unclassified LTRs	-	*0.13*	*0.69*	*1.71*	2.46	0.30	0.19	0.14	0.25	1.64
LINE/SINE	*0.41*	*0.56*	*1.04*	*0.47*	0.54	0.64	0.54	0.76	0.57	0.82
Unclassified RNA TEs	-	-	-	-	-	0.04	0.02	0.09	0.43	0.06
En-Spm	*0.55*	*0.74*	*1.04*	*0.65*	0.77	0.84	0.81	0.57	1.23	1.30
hAT	*0.06*	*0.12*	*0.24*	*0.11*	1.40	0.33	0.09	0.12	0.27	0.53
Mutator	*0.11*	*0.11*	*0.28*	*0.23*	0.49	0.18	0.20	0.23	0.24	0.57
RC/Helitron	*0.06*	*0.22*	*0.12*	-	-	0.25	0.16	0.09	0.18	-
PIF-Harbinger	-	-	*0.01*	*0.04*	-	-	-	-	-	-
Tc1-Mariner	-	-	-	*0.03*	0.05	-	-	-	-	-
Unclassified DNA TEs	-	-	-	-	0.21	0.48	0.63	0.42	0.17	-
rDNA	*0.67*	*1.74*	*1.34*	*1.36*	1.51	0.59	2.03	1.17	1.28	1.59
Satellites	*3.1*	*3.88*	*2.28*	*5.08*	14.41	13.45	11.23	12.92	2.59	9.40
Group II introns	-	-	-	-	0.09	-	-	-	-	-
Unclassified repeats	*7.57*	*4.57*	*6.06*	*3.71*	1.48	2.12	2.45	2.23	1.70	0.26
Total	*45.57*	*54.94*	*56.09*	*60.13*	68.68	67.34	76.59	78.53	55.42	57.69

Values in italics are reported from [11]. LINE, long interspersed nuclear element; LTR, long terminal repeat; SINE, short interspersed element; TE, transposable element.

We previously showed that variations in genome coverage have little effect on RepeatExplorer results [10]. To confirm this for the present study, we designed a combined dataset from all four Illumina-sequenced species that included 300,000 Illumina read pairs from *O. gracilis* (0.029X genome coverage). The repeat identification results are shown in Table 1. Analysis of *O. gracilis* individually or within a four-species dataset had no significant effect on repeat characterization: The total repeat content made up similar genomic proportion (68.68% and 67.34% for the individual and combined dataset, respectively), and the fractions of most individual repeat types also hardly differed. Thus, satellites made up 14.41% with the individual genome screening and 13.45% with the combined dataset. Detection of Gypsy retrotransposons showed greater differences between the two dataset (23.64% and 27.62% in the individual and combined dataset), probably due to a higher efficiency in Gypsy classification with a decrease in unclassified LTR element proportion (2.46% and 0.30% for the individual and combined dataset, respectively) compared to individual genome screening. The Gypsy content deduced from the combined dataset agreed with the value obtained just with 454 data, namely 28.34% [10]. Different from Gypsy retrotransposons, Copia retrotransposons are stably classified with the individual and combined dataset (21.63% and 20.52% for the individual and combined Illumina dataset), but appear underestimated with the 454 data (Table 1).

#### Comparison of the three tetraploid species with their diploid relative

We earlier suggested that tetraploidization, followed by genome downsizing, might have caused the observed enrichment in Gypsy retrotransposons and the loss of several repeat families in *O. gracilis* [10,11]. Here, we compared this species’ repeat content to that of its sister species *O. austrohispanica* (Figure 1) and the sister species to both of them, *O. densiflora*, as well as to a diploid relative, *O. rapum-genistae*. The three tetraploids have accumulated repetitive DNA (from 67.34% to 78.53%) compared to the diploid species (55.42%), and *O. austrohispanica* and *O. densiflora* turned out to contain even more repetitive DNA than *O. gracilis* (67.24% in *O. gracilis vs.* 76.59% in *O. densiflora* and 78.53% in *O. austrohispanica*). This repeat enrichment correlates with enrichment in Gypsy retrotransposons (27.62% in *O. gracilis vs.* 36.25% in *O. densiflora* and 39.64% in *O. austrohispanica*). The genomic proportions of other repeat types are similar among the three species; satellites make up 11.23% of *O. densiflora* genome, 12.92% of *O. austrohispanica* genome, and 13.45% of *O. gracilis* genome.

However, while polyploidization in Orobanchaceae may thus indeed be associated with repeat enrichment, especially of Gypsy elements, the genome of a diploid species, *O. rapum-genistae*, first analyzed here, has by far the highest Gypsy element concentration of any diploid Orobanchaceae studied so far ([10]; Table 1). Copia retrotransposons are underrepresented in this species (16.06% in *O. rapum-genistae* but 20.15% to 22.00% in the three tetraploids) and so are satellites (2.59% in *O. rapum-genistae* but 11.23% to 13.45% in the tetraploids). Indeed, *O. rapum-genistae* has one of the lowest genomic proportions of Copia elements in *Orobanche*.

To better understand the dynamics of Gypsy and Copia retrotransposons in the four species, we analyzed their elements in more detail using similarity searches against the GypsyDatabase [32]. The genome proportion of each clade is shown in Additional file 1. We were able to detect five major clades of Gypsy elements, but not the Ogre clade, which we had earlier shown to comprise a small fraction of the *O. gracilis* genome (0.08%, [11]). The genomic fractions of the Athila, Galadriel, and Reina clades obtained from the 454 and Illumina datasets of *O. gracilis* were congruent. By contrast, the Tekay and Tat elements are underrepresented in the Illumina dataset compared to the 454 dataset, and the centromeric retrotransposons (CRM) elements appear enriched in the Illumina dataset compared to the 454 data. Unclassified Gypsy elements constitute a larger fraction of the Illumina data than the 454 data, suggesting that they may partly correspond to underrepresented clades, especially Tekay. The increase in unclassified elements in the Illumina dataset is mostly due to shorter contig sequences. Interestingly, no unclassified Gypsy family is species specific.

The genomic proportions of unclassified Gypsy families found in all four species are similar and always small (Additional file 1: blue columns in second panel), ranging from 3.01% in *O. densiflora* to 3.70% in *O. austrohispanica*. Three unclassified families, however, are enriched in *O. rapum-genistae* in comparison with the tetraploids, making up 4.03% of the *O. rapum-genistae* genome and from 0.88% to 1.97% of the tetraploid genomes (Additional file 1: orange columns in second panel). The remaining unclassified families (taken together) appear enriched in the tetraploid *O. austrohispanica* (Additional file 1: yellow columns in second panel). We previously hypothesized that the polyploidization of *O. gracilis* may have led to enrichment in Tekay elements [11]. The results obtained here for the closely related tetraploids *O. austrohispanica* and *O. densiflora* support this hypothesis, with genomic proportions of Tekay and unclassified Gypsy elements even higher in these two species than in *O. gracilis*. The genomic proportion of Tekay elements in the diploid *O. rapum-genistae* (12.7%) overlaps that in the other diploid *Orobanche* species previously analyzed (9.74% to 12.52%, [11]).

The CRM elements comprise a similar genomic proportion among the three tetraploids (from 2.48% to 3.28%) but appear less abundant in the diploid *O. rapum-genistae* (1.07%), and Galadriel elements are enriched in *O. austrohispanica* compared to the three other species (Additional file 1). This enrichment is likely due to a recent burst of amplification of these elements in *O. austrohispanica* and probably explains the overrepresentation of Gypsy retrotransposons in this species in comparison with *O. densiflora*. The elements Tekay, CRM, and Galadriel all are chromoviruses, Gypsy retrotransposons that harbor a chromodomain, which is hypothesized to target the insertion of the elements into heterochromatin. The current results thus support our previous hypothesis [11] that polyploidization of *Orobanche* species may have led to the accumulation of chromoviruses.

Considering the Copia elements, we retrieved two groups of elements, the SIRE1/Maximus clade and a large class comprising the Tnt1, Angela, and TONT1 clades (Additional file 1). No Hopscotch families were detected in any of the four species, which is in agreement with our previous study on other species of *Orobanche* [11]. As regards the classified elements in *O. gracilis*, their estimated abundances are mostly congruent with the previous results obtained with 454 reads (Additional file 1), with the proportion of unclassified Copia elements notably lower than that of unclassified Gypsy elements. Two of the unclassified Copia families are enriched in the diploid *O. rapum-genistae* (making up 1% of its genome; Additional file 1), while the SIRE1/Maximus elements are enriched in the three tetraploid species, although the genome fraction they make up still overlaps that in other diploid *Orobanche* [11]. This pattern thus perhaps reflects a more efficient removal of SIRE1/Maximus elements in *O. rapum-genistae*, rather than an accumulation of these elements in the tetraploid clade.

#### Individual genome screening of the diploid *O. rapum-genistae*

To test whether the low abundance of Copia retrotransposons and satellites in *O. rapum-genistae* might result from undetected *O. rapum-genistae*-specific repeats, we analyzed 900,000 Illumina read pairs from *O. rapum-genistae* (corresponding to a genome coverage of 0.072X) as done for *O. gracilis*. This showed that the two approaches gave the same overall results, except in the case of three element types (Table 1). First, the Gypsy elements make up only 27.62% of the *O. rapum-genistae* genome with the individual genome screening, but 30.47% with the combined dataset. Second, satellites are more efficiently identified in the individual genome screening (9.40%) than in the combined dataset (2.59%), a result congruent with the genomic proportions of satellites observed in the tetraploids (previous section). Thirdly, Copia retrotransposon abundance was even lower in the individual genome screening than in the combined data, contrary to our expectation that Copia elements would be more efficiently fished out in the individual genome.

#### Phylogenetic signal in repeat family composition

**Figure 3:**
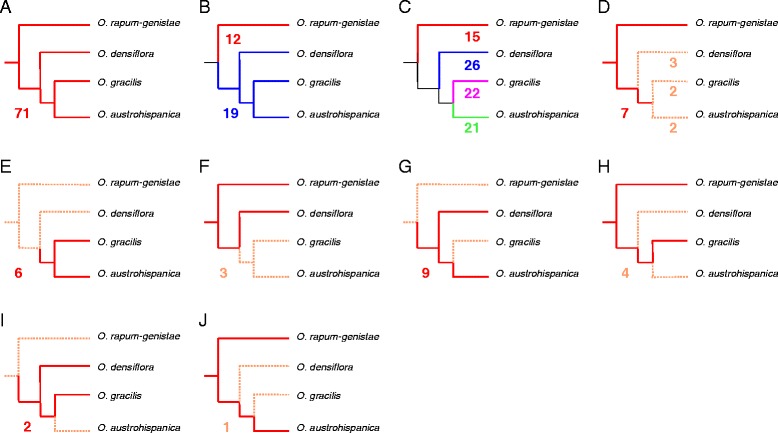
**Distribution of repeat families among the four Orobance species.** Each family is uniquely represented. Presence or overrepresentation is shown in continuous colored lines. Absence or underrepresentation is shown in dashed lines. For each distribution pattern, the number of families is given. **(A)** Families equally distributed among the four species. **(B)** In red: *O. rapum-genistae*-specific families. In blue: polyploid species-specific families. **(C)** Overrepresented families in either *O. rapum-genistae* (red), *O. gracilis* (blue), *O. densiflora* (purple), or *O. austrohispanica* (green). **(D)** Species-specific lost among seven widely distributed repeats. **(E)** Families specific to *O. gracilis* and *O. austrohispanica.* **(F)** Underrepresented or lost families in *O. gracilis* and *O. austrohispanica.* **(G)** Families specific to *O. densiflora* and *O. austrohispanica.* **(H)** Underrepresented or lost families in *O. densiflora* and *O. austrohispanica.* **(I)** Families specific to *O. gracilis* and *O. densiflora*. **(J)** Underrepresented or lost families in *O. gracilis* and *O. densiflora.*

Figure 3 shows the distribution of repeat families in the four species and demonstrates that phylogenetic relationships largely determine repeat distributions. Among the 218 identified repeat families, 71 are equally distributed among the four species, 12 are specific to *O. rapum-genistae*, and 19 are shared among the tetraploid species. Another large proportion of repeat families is overrepresented in individual species (15 in *O. rapum-genistae*, 21 in *O. austrohispanica*, 22 in *O. gracilis*, and 26 in *O. densiflora*). The 32 remaining repeat families are restricted to two or three species (Figure 3). The influence of phylogeny on the distribution of repeats was also observed in nine more distantly related Orobanchaceae [10] and fits with the general finding that repeat content tends to mirror phylogeny [37]. The similarity in the repeat families shared by the tetraploid species probably reflects that they already existed 6 to 7 Ma ago in the common ancestor of these species. The relatively high proportion of repeat families overrepresented in individual tetraploids, however, underlines the high dynamics of repeats and the variable effects of diploidization.

Our earlier hypothesis [10] that tetraploidy might have led to a burst of Gypsy retrotransposon activation in *O. gracilis* now seems less convincing, given that Gypsy retrotransposons make up a higher proportion of the diploid *O. rapum-genistae* genome than of the *O. gracilis* genome. Perhaps the genome of *O. rapum-genistae* is unusual (it also has the lowest proportions of Copia elements of all studied Orobanchaceae), but testing this will require broader species sampling. The sister species *O. austrohispanica* and *O. gracilis* differ by 0.3 pg in their genome size, corresponding to approximately 15% of the *O. gracilis* genome size, while the next closest species, *O. densiflora*, is intermediate in genome size, meaning that we cannot infer if genome size in *O. austrohispanica* increased or genome size in *O. gracilis* decreased after divergence from their common ancestor. It is clear, however, that TE dynamics in Orobanchaceae are highly unpredictable and that generalizations may be premature, agreeing with the cautionary conclusion of a recent review [1].

## Additional file

Additional file 1:
**Copia and Gypsy subclassification.** Histograms showing the genomic proportions of the Gypsy and Copia clades, with the corresponding estimates from Piednoël *et al.* [11] also shown.

## Abbreviation

TE: transposable elements
