# Supplementary figures and images for: Transposable elements in a clade of three tetraploids and a diploid relative, focusing on Gypsy amplification
Source: Mob DNA. 2015 Mar 25;6:5. doi: 10.1186/s13100-015-0034-8 (PMC4381496; doi:10.1186/s13100-015-0034-8)

## Gypsy

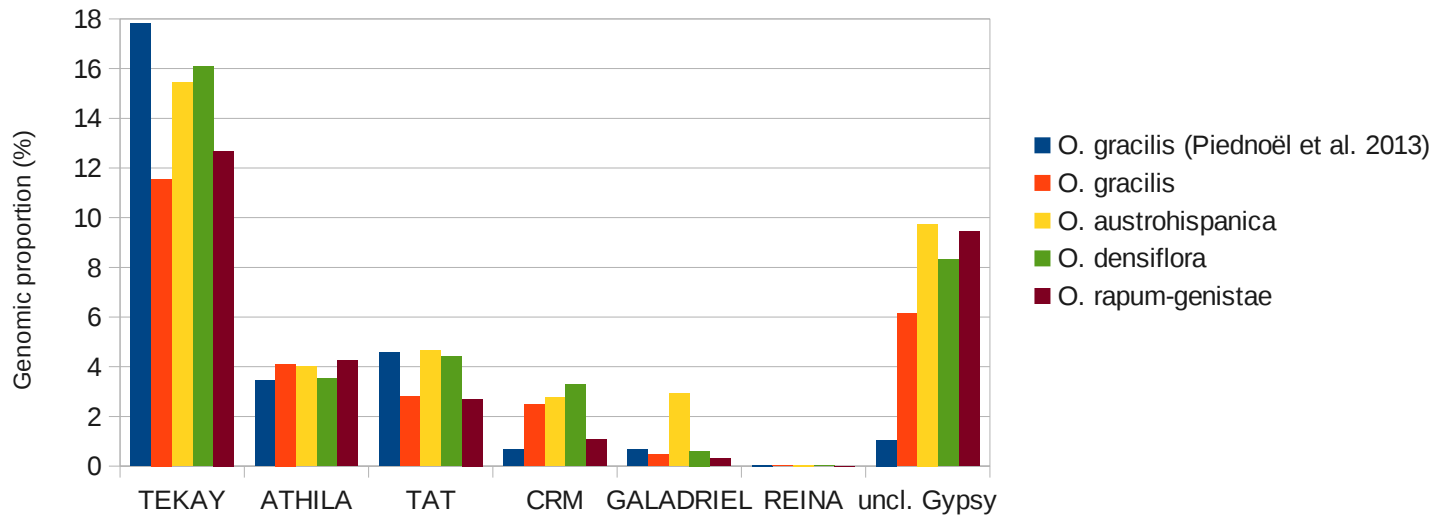

## Unclassified Gypsy

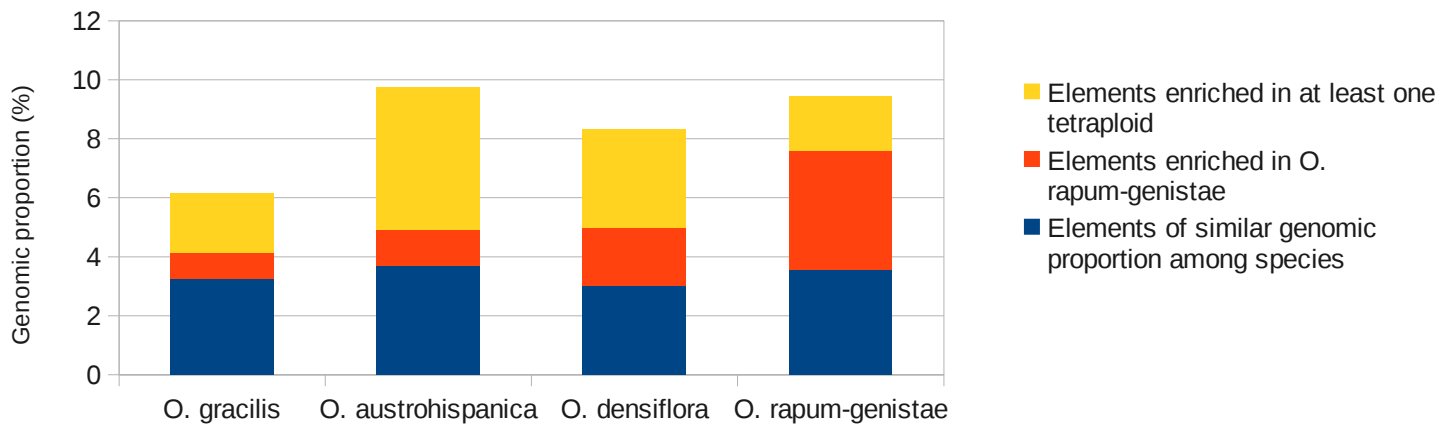

## Copia

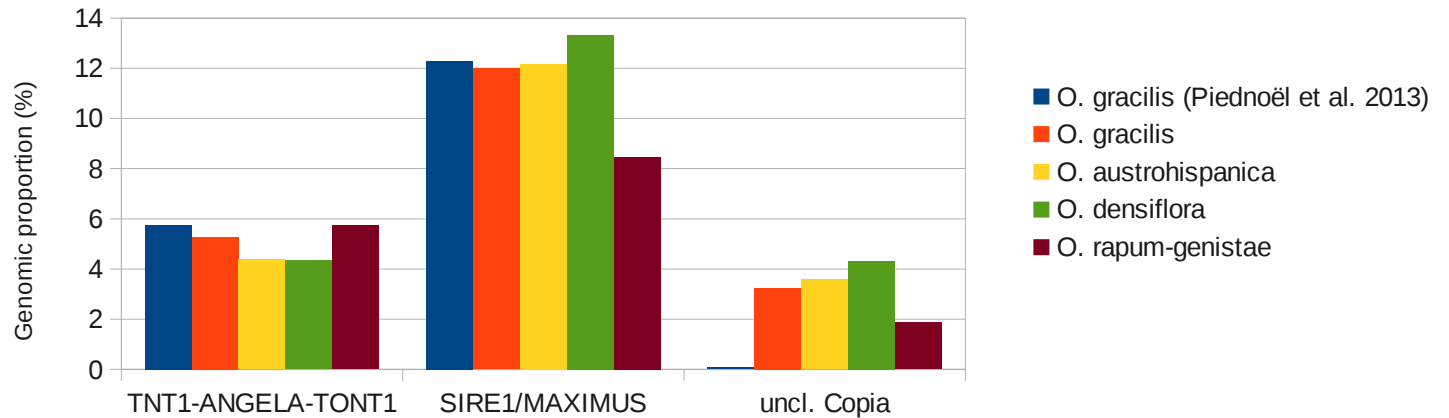

Supplement: Additional file 1: — Copia and Gypsy subclassification. Histograms showing the genomic proportions of the Gypsy and Copia clades, with the corresponding estimates from Piednoël et al. [11] also shown. [file 13100_2015_34_MOESM1_ESM.pdf]
